# Isolated metastasis of an EGFR-L858R-mutated NSCLC of the meninges: the potential impact of CXCL12/CXCR4 axis in EGFR_mut_ NSCLC in diagnosis, follow-up and treatment

**DOI:** 10.18632/oncotarget.24787

**Published:** 2018-04-10

**Authors:** Florian Lüke, Raquel Blazquez, Rezan Fahrioglu Yamaci, Xin Lu, Benedikt Pregler, Stefan Hannus, Karin Menhart, Dirk Hellwig, Hans-Jürgen Wester, Saskia Kropf, Daniel Heudobler, Jirka Grosse, Jutta Moosbauer, Markus Hutterer, Peter Hau, Markus J. Riemenschneider, Michaela Bayerlová, Annalen Bleckmann, Bernhard Polzer, Tim Beißbarth, Christoph A. Klein, Tobias Pukrop

**Affiliations:** ^1^ Department of Internal Medicine III, University Hospital Regensburg, Regensburg, Germany; ^2^ Chair of Experimental Medicine and Therapy Research, University of Regensburg, Regensburg, Germany; ^3^ Institute of Radiology, University Hospital Regensburg, Regensburg, Germany; ^4^ Intana Bioscience GmbH, Martinsried, Germany; ^5^ Department of Nuclear Medicine, University Hospital Regensburg, Regensburg, Germany; ^6^ Chair of Pharmaceutical Radiochemistry, Technische Universität München, Munich, Germany; ^7^ SCINTOMICS GmbH, Fuerstenfeldbruck, Germany; ^8^ Department of Neurology, University Hospital Regensburg, Regensburg, Germany; ^9^ Wilhelm Sander-Neurooncology Unit, University Hospital Regensburg, Regensburg, Germany; ^10^ Department of Neurology 1, NeuroMed Campus, Kepler University Hospital Linz, Linz, Austria; ^11^ Department of Neuropathology, University Hospital Regensburg, Regensburg, Germany; ^12^ University Medical Center Göttingen, Department of Medical Statistics, Göttingen, Germany; ^13^ University Medical Center Göttingen, Department of Hematology and Oncology, Göttingen, Germany; ^14^ Division Personalized Tumor Therapy, Fraunhofer Institute for Toxicology and Experimental Medicine, Regensburg, Germany

**Keywords:** NSCLC, brain metastasis, pentixafor PET/CT, CXCR4, fluorescence cross correlation spectroscopy

## Abstract

Brain and leptomeningeal metastasis (LMM) of non-small cell lung cancer is still associated with poor prognosis. Moreover, the current diagnostic standard for LMM often yields false negative results and the scientific progress in this field is still unsatisfying.

We present a case of a 71-year old patient with an isolated LMM. While standard diagnostics could only diagnose a cancer of unknown primary, the use of [^68^Ga]-Pentixafor-PET/CT (CXCR4-PET/CT, a radiotracer targeting CXCR4) and a liquid biopsy of the cerebrospinal fluid revealed the primary NSCLC. The detection of L858R-EGFR, a common driver mutation in NSCLC, enabled us to treat the patient with Afatinib and monitor treatment using [^68^Ga]-Pentixafor PET/CT. To estimate the impact of CXCR4 signaling and its ligands in NSCLC brain metastasis we looked at their expression and correlation with EGFR mutations in a primary and brain metastasis data set and investigated the previously described binding of extracellular ubiquitin to CXCR4.

In conclusion, we describe a novel approach to improve diagnostics towards LMM and underline the impact of the CXCL12/CXCR4 axis in brain metastasis in a subset of NSCLC patients. We cannot confirm a correlation of CXCR4 expression with EGFR mutations or the binding of extracellular ubiquitin as previously reported.

## INTRODUCTION

Brain metastasis (BM) and lepto-meningeal metastasis (LMM) of non-small cell lung cancer (NSCLC) is a severe clinical problem with significant impact on quality of life (QoL) and overall survival (OS). With the current local, intrathecal or systemic treatment approaches the 5-year overall survival is still only 15% in NSCLC patients with BM and less than 1% for patients with LMM [1]. At initial diagnosis about 7–10% of metastasized NSCLC patients already suffer from BM and overall 20–40% of the patients develop BM during the course of their disease [1]. In contrast to its clinical significance, the scientific efforts were barely noticeable in the past decades. Above all, patients with BM and specifically with LMM were categorically excluded from most clinical trials.

However, especially LMM requires more intensive scientific attention, because even diagnosing LMM is often difficult. This is especially true, when the disease presents in an occult fashion, if tumor material is difficult to reach for the acquisition of histologic material or the patient’s condition does not allow invasive procedures. Particularly for these scenarios, innovative, minimally-invasive diagnostic strategies are needed, especially in the era of “precision medicine” where a molecular diagnosis is obligatory for targeted therapy of NSCLC patients.

The current diagnostic standard of LMM patients are a neurological exam, a MRI of the brain and the neuroaxis and repeated classical cerebrospinal fluid (CSF) analyses, where cell count, glucose and protein levels and sometimes tumor markers are measured and a cytological investigation is performed. However, these classical methods are flawed by a significant diagnostic gap and can yield false negative results in LMM patients. Moreover, in most cases the current techniques are not suitable to detect specific genetic mutations due to the low count of tumor cells in the CSF. In this context, a liquid biopsy of the CSF is a reasonable option to improve diagnostics and eventually outcome of patients with LMM of NSCLC. By definition liquid biopsies, usually performed in, but not limited to blood samples, try to detect tumor specific genetic alterations outside a solid tumor or metastasis [2]. Current investigations demonstrate the diagnostic potential and power of CSF liquid biopsies, employing several different strategies. The CSF sample can be divided into a cellular and a cell free compartment. From both compartments, useful information can be extracted. By removal of the cellular compartment a relative enrichment of circulating tumor DNA (ctDNA) can be achieved in the cell free supernatant. CtDNA is more abundant in CSF than in plasma in patients with primary brain tumors and LMM and metastases that are connected to CSF [3]. This ctDNA can then be used to identify tumor specific mutations [4]. Another approach is to focus on the cellular compartment of CSF. The quality of the DNA is usually higher and less fragmented than in the cell free supernatant. The downside of this approach is the high amount of germline DNA contaminating the samples when total DNA is extracted [4]. A third strategy tries to overcome this limitation by enriching circulating/disseminated tumor cells, for example by selecting for the epithelial marker EpCAM [5]. With these techniques, an upfront evaluation of tumor biology/origin can be made, pointing clinicians into the right direction to consolidate their diagnosis.

Especially when staging patients with primary NSCLC [^18^F]FDG-PET/CT is currently the most sensitive tool and several guidelines incorporate [^18^F]FDG-PET/CT from the very beginning defining of the correct stage of the patients [6, 7]. [^18^F]FDG-PET/CT has proven its value, especially when dealing with so-called solitary pulmonary nodules (SPN). The literature reports a sensitivity of about 89% and a sensitivity of about 75% for SPNs as small as 8–10 mm [8]. On the other hand, there are some limitations to this technique. Firstly, it measures the glucose metabolism of the tissue as a surrogate parameter for its proliferative activity. Tumors with a low glucose metabolism might be missed. Secondly, metastases in tissues using glucose very intensively in their physiological state will be missed. This is especially true for BM, as the brain is almost solely dependent on glucose for energy generation [8].

In order to achieve a better diagnostic result, alternative radiotracers are of interest. Besides energy metabolism, there are numerous targets that are upregulated in NSCLC. One is the chemokine receptor 4 (CXCR4) which we previously demonstrated to be upregulated in BM of NSCLC and breast cancer patients [9]. Additionally, there is evidence that CXCR4/CXCL12 signaling is also a target of EGFR signaling. Tsai *et al.* showed that expression of L858R-EGFR in lung cancer cell lines, resulted in upregulation of CXCR4. This resulted in increased invasion in a modified Boyden chamber experiment as compared to wild-type expressing EGFR cell lines. They were also able to underline the connection of L858R-EGFR signaling to CXCR4 signaling by using an siRNA directed against CXCR4 and rescuing the invasive effect [10]. CXCR4 is also upregulated in malignant hematological diseases and involved in the regulation of the blood stem cell compartment [11, 12]. In line with these findings recently introduced CXCR4-targeted PET/CT imaging by means of the high affinity CXCR4 ligand [^68^Ga]-Pentixafor [13, 14] has been already proven to be complementary for diagnostic use in myeloma patients [15] compared to standard [^18^F]FDG-PET/CT [16]. Moreover, CXCR4 is a new immune-oncological target in the treatment of multiple myeloma, myeloid and solid cancers. New anti-CXCR4 antibodies or CXCR4 inhibitors are already in early clinical trials (e.g. NCT01359657, NCT01236144, NCT02765165). However, in patients with solid tumors the sensitivity of [^68^Ga]Pentixafor-PET/CT seems inferior to [^18^F]FDG-PET/CT in a small clinical study [17], although there is evidence, that for some patients there might be a clinical benefit [18].

CXCR4 is a G-protein coupled receptor with two known ligands, namely CXCL12 (SDF-1) and macrophage migration inhibitory factor (MIF) [19, 20]. CXCL12 is regarded the main ligand of CXCR4, while MIF binds to heteromers containing CXCR4, CD74 and CXCR2 [21]. Signaling of CXCR4 influences a variety of cellular pathways, including Gi-mediated inhibition of cAMP production, IP3-stimulated Ca^2+^ release, JAK/STAT signaling, PI3K/Akt and ERK signaling. Through these pathways CXCR4 signaling is involved in several crucial cell functions, including survival, growth, chemotaxis and also metastasis [22, 23]. Besides its physiological ligands, there is also evidence, that extracellular ubiquitin is a danger associated molecular pattern (DAMP) signal which might bind to and activate CXCR4 [24]. This finding could have enormous impact in particular in the tumor context, because of the high rate of cell damage and subsequent non-physiological amount of extracellular ubiquitin. Extracellular ubiquitin has further been shown to induce chemotaxis of monocytes and macrophages to a similar extent as CXCL12 [25]. Therefore, released ubiquitin from the necrotic center/cells of a tumor might be the most important source for CXCR4 activation and might additionally influence the composition of the tumor microenvironment. Since CXCR4 activation appears not to be limited to its physiological ligands [21], it is of great interest to identify the CXCR4 activators in the tumor to better stratify the use of “under investigation” CXCR4 inhibitors.

Here, we report a very uncommon case of LMM of a NSCLC patient which has led us to use liquid biopsy of CSF to identify an EGFR mutation and to explore the potential added value of [^68^Ga]-Pentixafor PET/CT in an [^18^F]FDG-PET/CT negative NSCLC.

## RESULTS

### Clinical report

**Figure 1:**
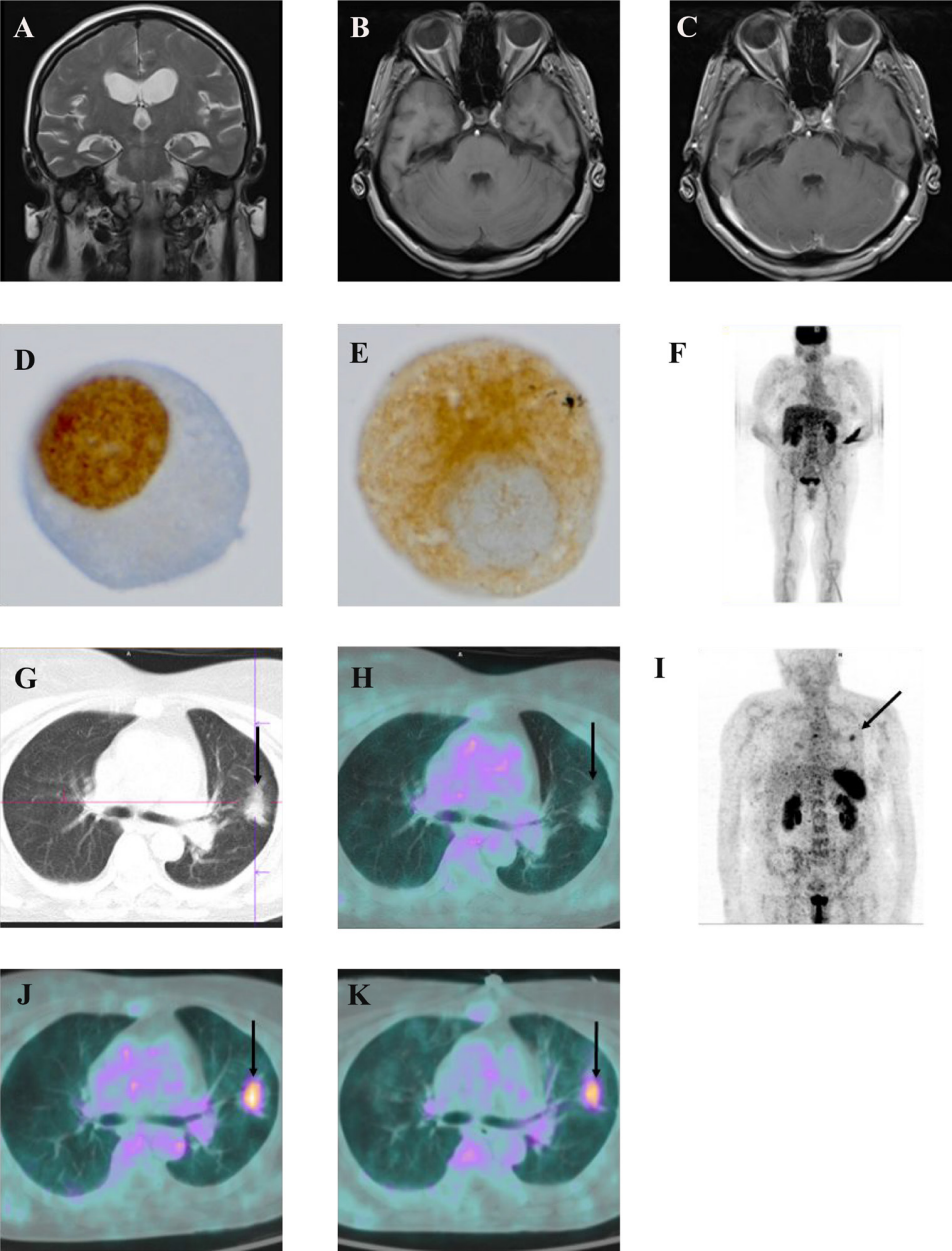
Clinical panel Coronal T2 MRI image showing signs of a CSF circulation disorder with ventriculomegaly and crowding of the gyri at the vertex with small sulci (**A**–**C**) axial T1 MRI images non contrast enhanced (B) and Gadobutrol enhanced (C) showing a representative section of the brain demonstrating no mengingeal enhancement and thus no MR-tomographic sign of meningeal carcinomatosis; (**D**–**E**) 1000x magnification of a single tumor cell in the cerebrospinal fluid, immunohistochemistry for TTF1 (**D**), immunohistochemistry for cytokeratin 18 (**E**) [^18^F]FDG-PET/CT scan showing no pathological pulmonal enhancement; (**F**): representative axial CT-image of the thorax showing an intrathoracal mass (arrow) (**G**) merged axial [^18^F]FDG-PET, CT image showing no tracer uptake in the intrathoracal mass (arrows); (**H**) [^68^Ga]-Pentixafor (CXCR4) PET scan showing a thoracal tracer enhancement in the left upper thorax (arrow), (**J**) representative axial merged [^68^Ga]-Pentixafor-PET, CT image showing tracer enhancement of the thoracal mass (arrow); (**K**) representative axial merged [^68^Ga]Pentixafor-PET, CT image 8 weeks after TKI treatment initiation, showing no significant reduction of tracer enhancement.

In July of 2015 a 71-year old woman was admitted to hospital with nausea, vomiting, tendency to fall, holocephalgia, partial memory loss and diplopic images. This set of symptoms had developed within 8 weeks. Additionally, she had been suffering from night sweats for the last 6 months and had experienced a moderate loss of weight of about 2 kg. Cranial MRI showed signs of a CSF circulation disorder (Figure 1A–1C, Supplementary Figure 1).

The analysis of CSF showed normal levels for cell count, protein and glucose. Intrathecal lactate was slightly elevated, hinting a non-specific intrathecal pathology. On neuropathological work-up, the conventional cytomorphological analysis revealed single atypical cells that proved to be cytokeratin 18 and TTF-1 positive on immunocytochemical stains (Figure 1D, 1E). With this staining result at hand, the primary lesion is most probably derived from an adenocarcinoma of the lung.

In a CT scan of the thorax an intrathoracic mass in the left upper lobe of the lung was detected (Figure 1G). An [^18^F] FDG-PET/CT however showed no tracer enhancement in this lesion (Figure 1F, 1H) and a transbronchial biopsy of the lesion did not reveal any sign of malignancy (data not shown).

### Single tumor cell mutational analysis in a CSF liquid biopsy reveals an EGFR-L858R missense mutation

**Figure 2:**
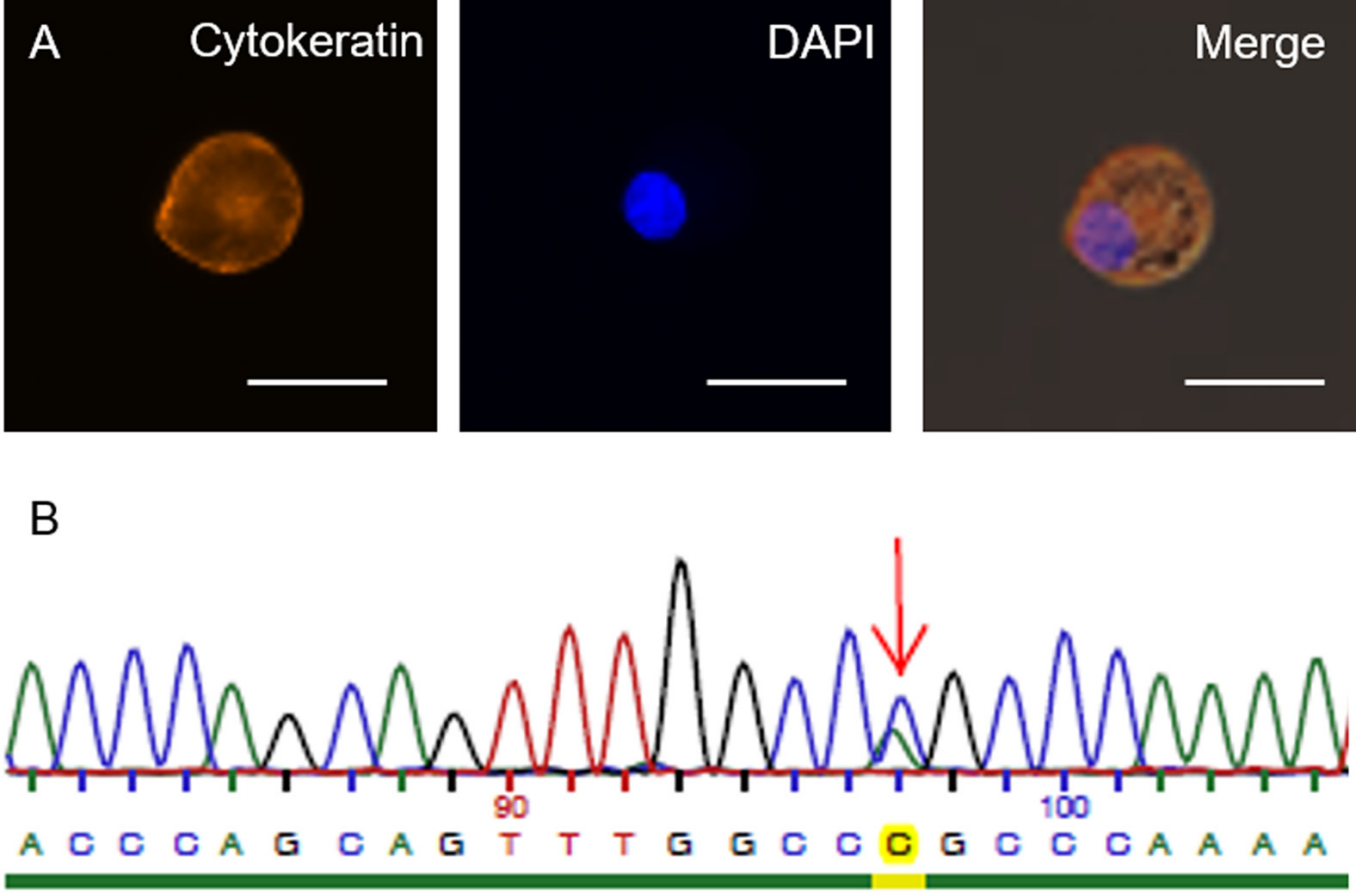
CSF liquid biopsy results (**A**) Disseminated tumor cell (DTC) isolated from cerebrospinal fluid of the patient. Staining against EpCAM and DAPI respectively; merged image of EGFR Exon 21 sequencing of an isolated DTC. Arrow indicates the position of the mutation using (L858R missense mutation) antisense sequencing primers (**B**). Scale bars represent 25 µm.

Next, we chose to analyze the peripheral blood and CSF by a liquid biopsy for the presence of circulating tumor cells (CTCs) from blood and disseminated cancer cells (DCCs) from CSF and to genetically characterize them by targeted sequencing. For this, cancer cells were enriched and detected using the CellSearch system that first enriches them by magnetic capture after anti-EpCAM ferrofluid labeling and visualizes cytokeratin-positive, CD45-negative tumor cells. Interestingly, tumor cells were only detected in CSF but not in peripheral blood (Figure 2A). In total, 14 DCCs were found in CSF, of which 10 were successfully isolated and individually subjected to whole genome amplification. Nine of these ten cells displayed high genome quality, as measured by the genomic integrity index GII [26], indicating that DCCs in CSF are more viable than CTCs in blood. Three cells with highest DNA quality (GII4) were selected for targeted sequencing of EGFR Exon 21 and we identified an EGFR-L858R missense mutation that is common in adenocarcinoma of NSCLC in two out of three DCCs (Figure 2B, Supplementary Figure 2).

### [^68^Ga]-Pentixafor-PET/CT (CXCR4-PET/CT) imaging identifies an [^18^F] FDG-PET/CT negative pulmonary lesion as primary tumor

Knowing that EGFR-L858R mutations have been shown to enhance the expression of CXCR4 [10], we performed a [^68^Ga]-Pentixafor-PET/CT (CXCR4-PET/CT) scan. Using this method, we identified the pulmonary lesion seen on the CT scans (Figure 1G) as the probable primary tumor. The meningeal affection that initially led to hospitalization of the patient showed no increased [^68^Ga]-Pentixafor-PET/CT uptake, probably because the patient had already received 2 courses of intrathecal chemotherapy up to this point (Figure 1I, 1J). After having established a precise diagnosis the patient was treated with Afatinib 40 mg for 8 weeks. Afatinib is a second generation tyrosine kinase inhibitor used for treatment of EGFR mutated NSCLC. Its ability to cross the blood brain barrier made it the drug of choice for our patient. The following [^68^Ga]-Pentixafor-PET/CT was used for therapy monitoring and demonstrated a persistent tumoral uptake of ^68^Ga-Pentixafor and no change in the size of the pulmonary target lesion according to RECIST criteria, suggesting no metabolic sign of treatment response and radiologically stable disease (Figure 1K). Because of treatment related side effects, a Karnofsky Performance Status (KPS) of 50%, lack of clinical improvement and no changes in ^68^Ga-Pentixafor uptake, we discontinued Afatinib.

### EGFR mutation does not necessarily lead to enhanced CXCR4 expression in lung cancer primaries

**Figure 3:**
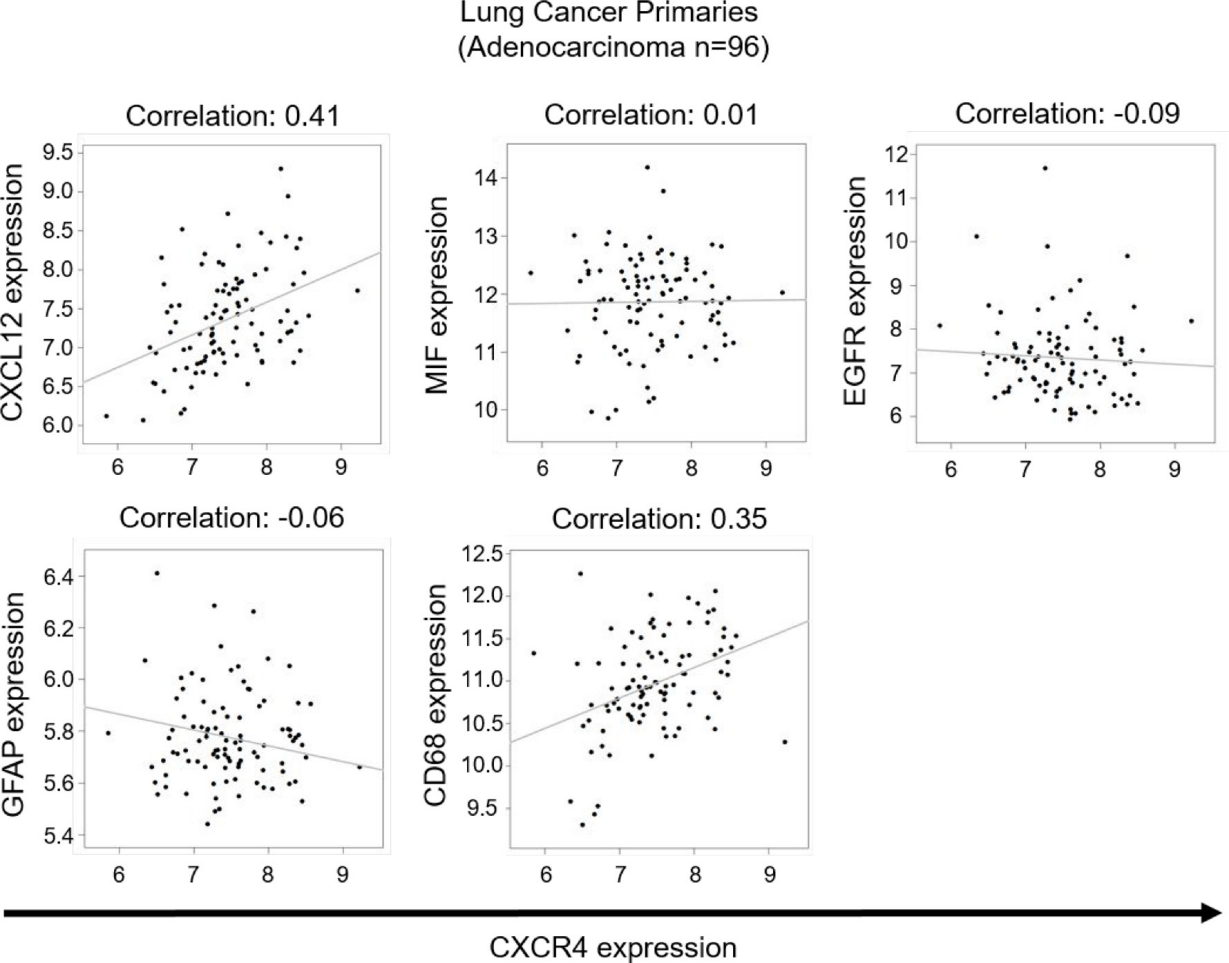
Correlation studies on lung cancer primaries Analysis of microarray data from lung adenocarcinoma primaries. Scatterplots represent expression of 96 samples on the comparison between CXCR4 and 5 genes (CXCL12, MIF, EGFR, GFAP, CD68), the Pearson correlation coefficients between them are shown as fitting slop lines on each panel. *Q*-values represent the adjusted *p*-value modified for multiple testing using the Bonferroni-approximation.

Because of this experience and the potential dependency of CXCR4 expression on EGFR-stimulation [10], we asked if activating EGFR mutations in NSCLC primaries generally correlate with elevated CXCR4 expression. Using publicly available data from previously published Microarray analyses we tested our hypothesis *in silico*. Firstly, we correlated the expression of CXCR4 in a Microarray data set of NSCLC primaries with the EGFR wildtype (*n* = 192) and EGFR mutated (*n* = 30) cohort, which resulted in no significant difference (*q* = 0.8) (Table 1). Secondly, using data from the same gene set, we performed a bioinformatics analysis to assess a correlation between CXCR4 and EGFR gene expression levels. We could not show any significant correlation between these two parameters (Figure 3, Table 3).

**Table 1:** Tabular results of a correlation analysis between EGFR wt and EGFR mut lung adeno and squamous cell carcinoma primary samples

Gene	logFC	Average Expression	mean.EGFRmt	mean.EGFRwt	*p*-Value	*q*-Value
CXCL12	−0.06	7.33	7.28	7.34	0.67	0.93
GFAP	0.01	5.74	5.75	5.74	0.74	0.95
MIF	−0.27	12.14	11.91	12.18	0.08	0.59
EGFR	−0.19	7.45	7.29	7.48	0.32	0.80
CD68	−0.10	10.75	10.66	10.76	0.48	0.87
CXCR4	−0.14	7.40	7.28	7.42	0.32	0.80

LogFC representing the Log_2_ fold change in expression.

**Table 3:** Tabular results of a correlation analysis between CXCR4 expression and CXCL12, MIF, EGFR, GFAP, CD68 in lung adenocarcinoma primary samples

Primary lung cancer sample	Gene expression compared to CXCR4	Correlation coefficient	*p*−Value	*q*−Value
Adeno−carcinoma (*n* = 96)	CXCL12	0.41	1.79 × 10^−5^	5.3 × 10^−4^
	MIF	0.01	0.86	1
	EGFR	−0.09	0.34	1
	GFAP	−0.06	0.55	1
	CD68	0.36	0.00029	8.9 × 10^−3^
NSCLC (*n* = 220)	CXCL12	0.49	1.05 × 10^−14^	3,14 × 10^−13^
	MIF	0.01	0.84	1
	EGFR	−0.055	0.41	1
	GFAP	−0.01	0.83	1
	CD68	0.52	1.61 × 10^−16^	4.84 × 10^−15^

**Figure 4:**
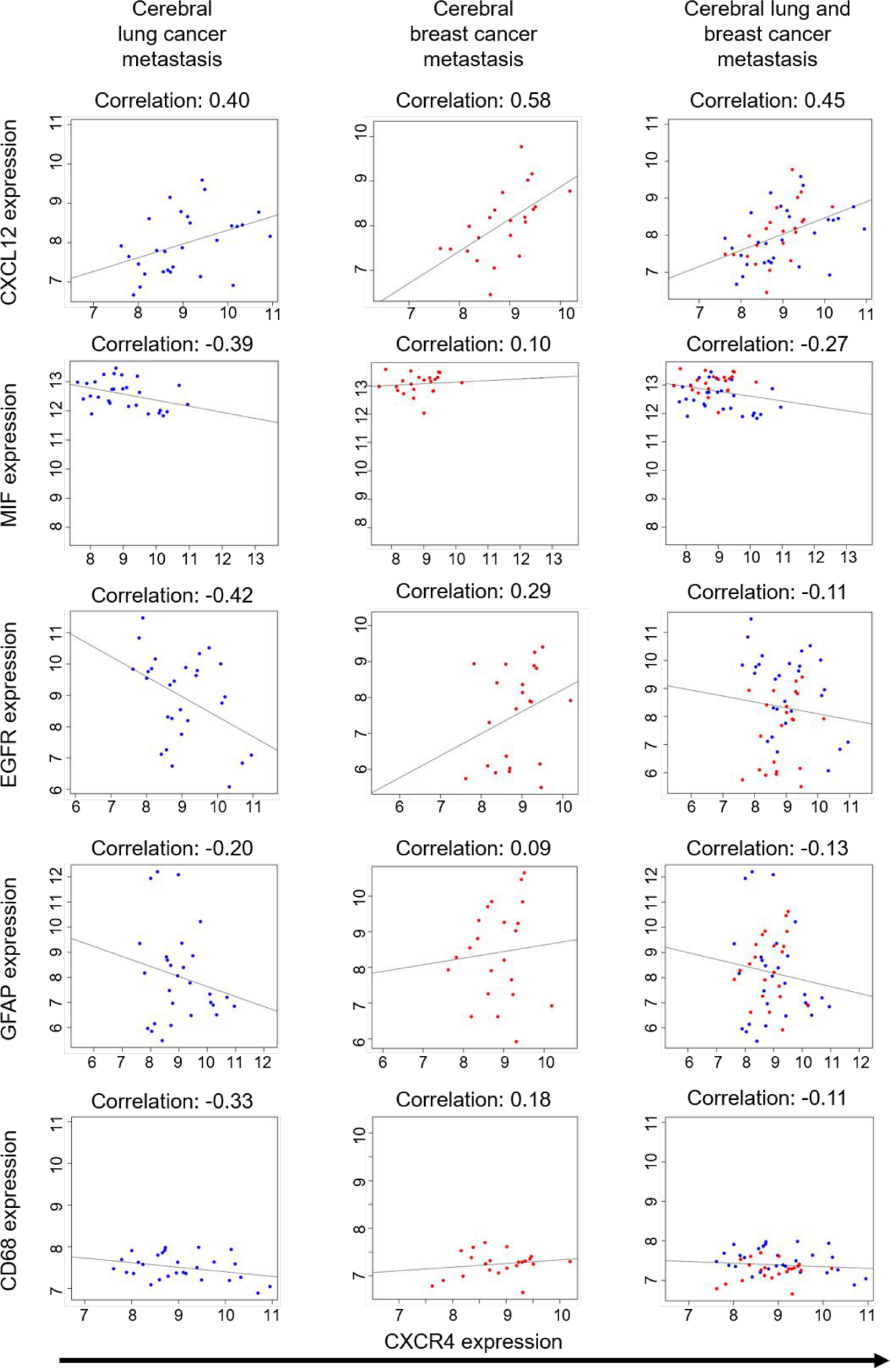
Correlation studies on breast and lung cancer brain metastases Analysis of Affiymetrix Microarray data from 28 lung cancer metastatic samples and 22 breast cancer metastatic samples. Scatterplots represent expression of the samples on the comparison between CXCR4 and 5 genes (CXCL12, MIF, EGFR, GFAP, CD68), the Pearson correlation coefficients between them are shown as fitting slop lines on each panel. *Q*-values represent the adjusted p-value modified for multiple testing using the Bonferroni-approximation.

On the other hand, the analysis of the physiological ligands, namely CXCL12 and MIF, showed a significant correlation between CXCR4 and CXCL12 expression (Figure 3, Table 3). There was also a significant correlation between CD68 and CXCR4 expression in these samples (Figure 3, Table 3). With the central nervous system (CNS) being a compartment with considerably reduced immune reaction, we hypothesized that there might be a different expression pattern in BM and repeated our *in silico* testing with samples from brain metastases. Using Affymetrix expression data from 29 lung and 22 breast carcinoma brain metastases [9], we again were unable to find a correlation between CXCR4 and EGFR expression (Figure 4, Table 2). Interestingly, again CXCR4 expression correlated with the expression of CXCL12 (Figure 4, Table 2).

**Table 2:** Tabular results of a correlation analysis between CXCR4 expression and CXCL12, MIF, EGFR, GFAP, CD86 in brain metastastatic samples

Metastatic sample	Gene expression compared to CXCR4	Correlation coefficient	*p*-Value	*q*-Value
Lung cancer (*n* = 28)	CXCL12	0.405	0.033	0.491
	MIF	−0.387	0.042	0.627
	EGFR	−0.418	0.027	0.404
	GFAP	−0.195	0.319	1
	CD68	−0.327	0.089	1
Breast cancer (*n* = 22)	CXCL12	0.577	0.005	0.074
	MIF	0.097	0.667	1
	EGFR	0.288	0.193	1
	GFAP	0.087	0.700	1
	CD68	0.181	0.420	1
Lung cancer and breast cancer combined	CXCL12	0.449	0.001	0.016
	MIF	−0.270	0.058	0.869
	EGFR	−0.110	0.447	1
	GFAP	−0.130	0.368	1
	CD68	−0.110	0.447	1

### Extracellular ubiquitin does not co-localize with CXCR4 and does not compete with CXCL12 for CXCR4 binding in a FCCS approach

**Figure 5:**
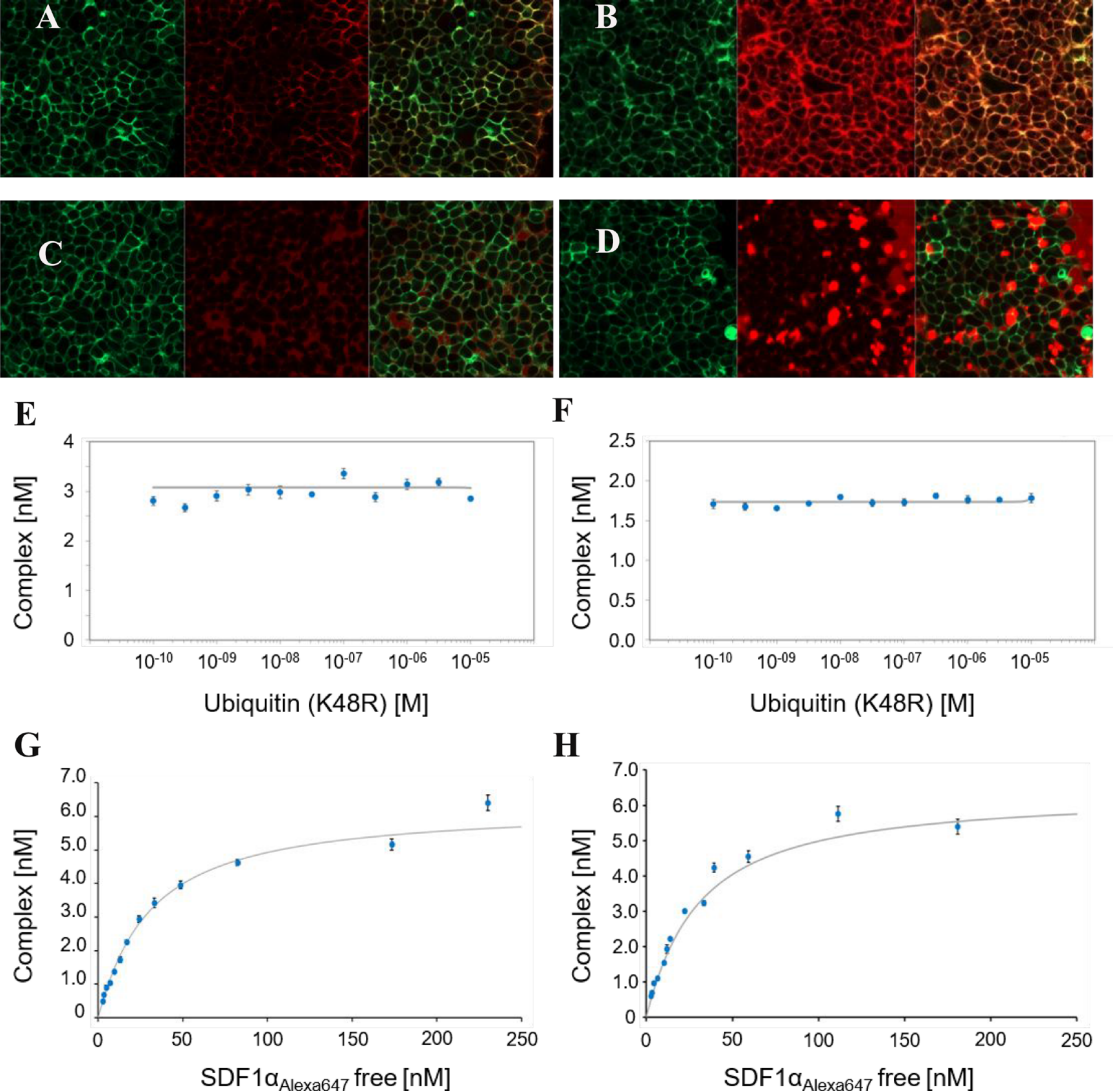
FCCS studies (**A**, **B**) Binding of 50 nM Alexa647 (red color) labeled CXCL12 to CXCR4-GFP (green color) expressing HEK293-cells 2 minutes (A) and 20 minutes (B) after additon of CXCL12 (left panel showing GFP labeled CXCR4, middle panel showing Alexa647 labeled CXCL12, right panel showing the merged image); (**C**, **D**) binding of 200 nM Alexa 647 labeled ubiquitin (red color) to CXCR4-GFP expressing HEK293-cells (green color) 2 minutes (C) and 20 minutes (D) after addition of ubiquitin; (left panel showing GFP labeled CXCR4, middle panel showing Alexa647 labeled Ubiquitin, right panel showing the merged image) (**E**) competition binding assay of eGFP labeled CXCR4, Alexa 647 labeled CXCL12 and unlabeled His_6_-ubiquitin(K48R), IC_50_ > 10 µM, K_i_ > 10 µM; (**F**) competition binding assay of eGFP labeled CXCR4, PEG4-DY647 labeled anti-CXCR4mAb and unlabeled His_6_-ubiquitin(K48R), IC_50_ > 10 µM, K_i_ > 10 µM; (**G**, **H**) saturation binding assay of eGFP labeled CXCR4 and Alexa 647 labeled CXCL12 without ubiquitin (G) and 80 µM unlabeld ubiquitin (H).

Finally, we were interested in possible alternative ligands of CXCR4. We looked at extracellular ubiquitin in closer detail because it has been shown to be a DAMP signal for tissue damage and hypoxia and it plays a role in B-cell and monocyte function [27, 28]. This might be especially interesting in tumors, as they often contain a hypoxic, necrotic center region where one would suspect big amounts of extracellular ubiquitin. Ubiquitin is a highly conserved and ubiquitous intracellular protein that is released into the extracellular space in cases of tissue damage [24]. Recently extracellular ubiquitin was described as alternative ligand for CXCR4 and therefore as an endogenous DAMP signal with CXCR4 being an allosterically modulated DAMP receptor [29–32]. To test binding of ubiquitin to CXCR4 we used fluorescence-labeled CXCL12, fluorescence-labeled ubiquitin and GFP-tagged CXCR4 in HEK293 cells. While CXCL12 co-localized with CXCR4 (Figure 5A, 5B), the labeled ubiquitin did not (Figure 5C). It rather showed a diffuse intracellular localization after 20 minutes of incubation (Figure 5D). Next, we tested if ubiquitin is a competitive ligand to CXCR4 by a FCCS approach. Our results show that ubiquitin can neither displace CXCR4’s innate ligand CXCL12 nor a CXCR4 monoclonal antibody from the chemokine receptor (Figure 5E, 5F). This suggests no direct competition for the CXCL12 binding site. Since this does not rule out an allosteric mechanism of ubiquitin action on CXCR4 we proceeded to test the binding affinity of CXCL12 in the presence and absence of ubiquitin. Yet again we could not find a change in receptor binding kinetics (Figure 5G, 5H).

## DISCUSSION

This uncommon case report of a [^18^F] FDG-PET/CT negative NSCLC patient, who presented with isolated LMM illustrates the current limitations of the standard of diagnostics for LMM patients. Moreover, it also emphasizes that particularly patients with an unusual course of NSCLC do profit more from innovative diagnostic techniques, like liquid biopsy of CSF and innovative imaging methods. However, these rare courses of NSCLC are usually not considered for clinical trials. Thus, the current study designs with very strict inclusion criteria harbor a risk that a potential added value for these patients with innovate techniques will be completely overlooked. In contrast, our further bioinformatics analyses also taught us that general conclusions from case reports should be handled carefully [10]. We assumed, that there was a link of CXCR4 expression and the mutation status of EGFR, and had therefore implemented Pentixafor-PET/CT from the beginning. Although the [^68^Ga]-Pentixafor-PET/CT revealed the probable primary tumor, the bioinformatics analyses of gene expression data could not support our assumption that CXCR4 expression and the mutation status of EGFR are linked. However, our data do not completely rule out a more complex interaction of CXCR4 and EGFR signaling. Nevertheless, the [^68^Ga]-Pentixafor-PET/CT identified the primary tumor in our case and was very useful in treatment follow-up and clinical decisions made. Yet, we aimed to gain a better idea about the clinical circumstances in which a [^68^Ga]-Pentixafor-PET/CT in solid tumors could help to diagnose [^18^F]FDG-PET/CT negative tumors. Therefore, we analyzed selected genes which could be at least theoretically linked to the expression of CXCR4. This analysis revealed that only the physiological ligand CXCL12 correlated with the CXCR4 expression in all tested data sets of the tested genes.

To estimate the potential impact of deregulated CXCR4 activity in tumor tissue, we investigated the recently described alternative DAMP ligand, ubiquitin [30]. Ubiquitin is, due to cell death and necrosis, present in non-physiologically high, extracellular concentrations in tumor tissue and could therefore be the relevant ligand for CXCR4 in a tumor. However, our binding studies of extracellular ubiquitin to CXCR4 showed no direct interaction of CXCR4 with this molecule. We also could not show an allosteric modulation of CXCL12 binding affinity to CXCR4 by our FCCS studies. This is in contradiction with results of Saini *et al.*, who showed that CXCL12 could replace fluorescently labeled ubiquitin from cells in a CXCR4 dependent fashion [30]. We hypothesize that there might be CXCR4 signaling related to extracellular ubiquitin, i.e. by recruiting a second protein to form heterodimers with CXCR4. For example, CXCR4 has been shown to form heterodimers, i.e. with ACKR3 [33]. The analysis of CXCL12 expression could be a very interesting candidate for future stratification not only when using [^68^Ga]-Pentixafor-PET/CT, but also when considering potential inclusion criteria for CXCR4 inhibitor trials. Recently, beta emitter labeled analogs of CXCR4-ligands ([^177^Lu]/[^90^Y] Pentixather) have been tested as a novel therapeutic approach in patients with hematological neoplasm (i.e. multiple myeloma). The [68Ga]-Pentixafor-PET/CT in our patient showed a high tracer uptake, so Pentixather could have had a therapeutic benefit, in principle. However due to its strong myeloablative effect CXCR4-directed radionuclide therapy seems not to be of clinical value in this tumor entity [34].

Moreover, our patient’s case also demonstrates the potential power of tumor cell enriching liquid biopsies of CSF in LMM patients. When looking at patients with isolated LMM, even if disseminated tumor cells are found in a lumbar puncture, the underlying tumor entity can be precisely identified in a standard cytology assessment, as we demonstrated above. Liquid biopsies using ctDNA and DNA extracted from the cellular compartment do bear limitations: they work best in CSF-cytology positive patients [4]. This still adds significant diagnostic value, but the sensitivity to detect LMM in the first place remains at the level of a single lumbar puncture (∼50%). By using tumor cell enriching techniques, as in our case, the sensitivity can be significantly enhanced. This is also underlined by a small study in NSCLC patients, which directly compared the sensitivity of MRI (47.6%), classical CSF-cytology (57.1%) and CellSearch enriched DTC analysis (95.2%) of CSF for diagnosing LMM. Thus, liquid biopsies can help to identify primary tumor origin in patients, that otherwise would have been classified as cancer unknown primary (CUP).

Taken together, since LMM is in most cases a prognosis limiting condition [35] more scientific efforts should be undertaken to implement and validate innovative techniques. In the future, we need such highly precise tools in diagnosing and treating these patients, in particular if they present with an uncommon clinical course. In this context, the liquid biopsy of the CSF and the [^68^Ga]-Pentixafor-PET appear to be very promising.

## MATERIALS AND METHODS

### Patients and CTC enrichment and detection using the CellSearch^®^ assay

A blood sample and cerebrospinal fluid were obtained from the patient based on principles set out in the WMA Declaration of Helsinki and approved by the Ethics Committee of University of Regensburg. Enrichment and detection of CTCs was performed using the CellSearch system (Riethdorf *et al.*, 2007). Briefly, 6 ml of CSF and 10 ml of blood were obtained and transferred into a CellSave tube (Veridex Inc.). The CellSearch Epithelial Cell Test (Veridex Inc.) was applied for enrichment and enumeration of circulating tumour cells (CTCs) from blood and disseminated cancer cells (DCC) from CSF according to the instruction from the manufacturer. CTCs are captured from 7.5 ml of blood by anti-epithelial cell adhesion molecule (EpCAM)-antibody-bearing ferrofluid and subsequently checked for positivity or negativity for cytokeratin, the leukocyte common antigen CD45 and 4´,6-diamidino-2-2phenylindole (DAPI) staining to ensure the integrity of the nucleus. For enrichment of DCC from CSF we modified the protocol. After puncture, the sample was transferred to a CellSave tube. After a short incubation the sample was transferred to a CellSearch Conical Centrifuge tube and sample preparation using the CellTrack Autoprep - system was started and run similar to a control sample and analysed by CellTracks AnalyzerII.

### Isolation of CTCs and WBCs by micromanipulator and microscope

After epithelial cell enrichment cells from blood or CSF were extracted from CellSearch cartridge using a 200 µl gel-tip pre-rinsed in PBS-BSA 2% and transferred to a new protein LoBind 1.5-ml tube (Eppendorf, Germany). The cartridge was subsequently washed two times using 325 µl of PBS (PAN Biotech) and repeatedly pipetting against the inner surface. The complete fluid was transferred to the 1.5-ml sample tube and centrifuged at 1,000 g for 5 min in a swinging-bucket rotor centrifuge. After discarding the supernatant by pipetting, 1 ml of PBS was added and the tube again centrifuged at 1,000 g for 5 min in a swinging-bucket centrifuge. Again the supernatant was removed, and the pellet was finally resuspended in 200 µl PBS. The sample was then loaded one field of a BSA (20 mg/ml in H_2_O, Sigma-Aldrich) covered glass slide (Nunc^*®*^ Lab-Tek^®^ Chamber Slide^™^ system 8 wells, Thermo Fischer) and screened by fluorescence microscopy (IX81, Olympus, Germany). Using the Cy3- and DAPI fluorescence filter the CTCs/DCCs were identified, using the APC and DAPI fluorescence filters the WBCs were identified and both by micromanipulation using a glass capillary of ≈ 30-µm diameter pre-rinsed with FCS, transferred to a new BSA covered field with 200 µl PBS. Here, by pipetting of 1 µl, the cells were singly picked into individual 200 µl tubes (Nerbe plus, Germany) including 2 µl of Proteinase K digest mix (first step of Whole genome amplification).

### Whole genome amplification

The method is based on a published adaptor-ligation-mediated whole genome amplification protocol (Klein *et al.*, 1999, 2002) and has become commercially available as Ampli1-kit (Silicon Biosystems). In brief, after the Proteinase K digest (10 h 42° C, inactivation for 10 min at 80° C), single cell DNA was digested by MseI restriction endonuclease (3 h 37° C, 65° C for 5 min of inactivation). Adaptor formation by pre-annealing of ddMSE primer and LibI primer was performed with a starting temperature of 65° C and shifted down to 15° C with a ramp of 1° C/min. At 15° C, 1 µl of ATP (10nM) and 1 µl of T4-DNA-Ligase (5 units) were added and primers and DNA fragments were ligated overnight. Resulting in 50 µl of WGA product the primary amplification was started.

### Quality control assay

For the multiplex PCR, 1 µl WGA template was used in 10 µl of a water-based mastermix containing 1x FastStart PCR Buffer (including MgCL_2_), 200 nM dNPTs, 0.5 U FastStart Polymerase and 4 µg BSA (all consumables Roche Diagnostics GmbH, Germany). The eight primers (CK19, TP 53 Exon 2/3, D5S2117, KRAS) were each used in an end concentration of 0.4 µM. PCR was started with a first step at 95° C for 4 min, followed by 32 cycles of 95° C for 30 s, 58° C for 30 s and 72° C for 90 s, and final elongation step of 7 min at 72° C. To determine the genome integrity index, PCR products were visualized on a 1.5 % agarose gel. The protocol of the multiplex PCR assay is based on the commercially available *Ampli*1™ QC kit (Silicon Biosystems, Italy).

### Analysis of microarray datasets

Data were analyzed analogous to [9]. Briefly Affymetrix data sets of 28 brain metastasis samples from primary adenocarcinomas of the lung (GSE 14108) and 22 brain metastasis samples from primary breast cancers (GSE 14017, GSE 14018) were subjected to correlation analysis using the free statistical software R (http://www.r-project.org). The data sets are publicly available on Gene Expression Omnibus (GEO, https://www.ncbi.nlm.nih.gov/geo/query/acc.cgi). The metastases used to generate this data set were profiled and compared by the expression level of over 400 cytokines. If not otherwise specified correlation coefficients and *p*-values were calculated according to the Pearson correlation method supplied by R. *Q*-values given represent corrected *p*-values according to the Bonferroni approximation, to adjust for errors caused by multiple testing. The Bonferroni method was used, for its conservative calculation profile. It usually yields higher *q*-values than other methods used. While disposing some results as insignificant, the probability of a false positive result is greatly reduced. Significance threshold for *p*- and *q*-values alike is <0.05. Correlation analysis of primary lung cancer samples were performed using data from [36]. The source data are available under http://stm.sciencemag.org/content/5/209/209ra153/tab-figures-data and http://www.uni-koeln.de/med-fak/clcgp/.

### Fluorescence cross correlation spectroscopy (FCCS) analysis

Reagents were obtained from Anatrace (Maumee, OH, USA), AppliChem (Darmstadt, Germany), Carl Roth (Karlsruhe, Germany), or SigmaeAldrich (St. Louis, MO, USA) unless stated otherwise. Unlabeled compounds were purchased from Tocris (Bristol, UK) or Abcam (Cambridge, UK) with the exception of (¬)-norepinephrine and SR48692 (SigmaeAldrich), SR 142948A (Santa Cruz Biotechnology, Santa Cruz, CA, USA), neurotensin (8e13) (NT; AnaSpec/MoBiTec, Göttingen, Germany), and TC 14012 (Cayman Chemical, Ann Arbor, MI, USA). Anti-CXCR4 monoclonal antibody clone 44708 (murine IgG2A; cat. no. MAB171) was acquired from R&D Systems (Minneapolis, MN, USA). Unlabeled recombinant CXCL12/SDF-1a was obtained as a gift from Annette Beck-Sickinger (Institut für Biochemie, Universität Leipzig, Germany). Human synthetic stromal cell-derived factor 1 (SDF1a) labeled specifically on residue Lys64 with AlexaFluor 647 (henceforth “SDF1a-AF647”; cat. no. CAF-11) was acquired from Almac (Craigavon, UK).

FCCS analysis was carried out as described in [37]. Briefly, CXCR4 was expressed as C-terminal fusion to GFP in HEK293 cells. Membranes were prepared by sonification on ice. Cell debris was removed and membranes harvested from the cleared lysate by subsequent centrifugation at 21.000 g. The receptors were solubilized by incubation in detergent mix of DDM (n-dodecyl β-D-maltoside)/CHAPS/CHS (cholesteryl hemisuccinate). Insolubilized membrane material was removed by centrifugation at 100.000 g. Solubilized GPCRs were directly used for FCCS binding assays.

FCCS measurements with samples at equilibrium were performed with a ConfoCor2 FCS unit connected to an Axiovert 100M equipped with a C-Apochromat 40× water immersion lens, NA 1.2 (Carl Zeiss, Jena, Germany) whereas FCCS-data for binding kinetics were acquired on an Insight plate reader (Evotec Technologies, Hamburg, Germany) fitted with a U-Apo300 40× water immersion lens, NA 1.15 (Olympus, Germany). The kinetics was monitored by FCCS over the course of 20–60 min. For more detailed information see Supplementary methods.

### Preparation of [^68^Ga]-pentixafor

[^68^Ga]-Pentixafor was synthesized in a fully automated procedure on a Scintomics GRP module (SCINTOMICS GmbH, Germany) connected to a ^68^Ge/^68^Ga generator (iThemba Labs, South Africa) and equipped with a disposable single-use cassette kit (ABX GmbH, Germany) using a standardized labeling sequence [38]. For more detailed information see Supplementary Methods.

### [^18^F]FDG PET/CT, ^68^Ga-Pentixafor-PET/CT

[^18^F]FDG -PET/CT imaging was performed using a Biograph 16 PET/CT scanner (CTI-Siemens, Erlangen, Germany). After a fasting period of at least 4 h, 3 MBq [^18^F]FDG per kilogram body weight were injected intravenously. After a waiting period of 60 min post injection the PET/CT acquisition was performed. PET images (slice thickness 5 mm) were corrected for random coincidences, decay, scatter and attenuation and reconstructed iteratively using the ordered subsets expectation maximization algorithm (OSEM) with 4 iterations and 8 subsets. [^68^Ga]-Pentixafor-PET/CT was performed the same way with an activity of 1.5 MBq [^68^Ga]-Pentixafor-PET/CT per kilogram body weight without fasting.

## SUPPLEMENTARY MATERIALS FIGURES



## Abbreviations

EGFR: Epithelilal Growth Factor Receptor
NSCLC: Non Small Cell Lung Cancer
LMM: Leptomeningeal metastasis
CUP: Cancer Unknown Primary
PET: Positron Emission Tomography
CT: Computed Tomography
TKI: Tyrosine kinase inhibitor
CSF: Cerebrospinal Fluid
BM: Brain Metastasis
OS: Overall survival
ctDNA: Circulating tumor DNA
DNA: Desoxyribonuclear Acid
EpCAM: Epithelial Cell Adhesion Molecule
SPN: Solitary Pulmonary Nodule
CXCR4: Chemokine Receptor 4
CXCL12: Chemikine Receptor Ligand 12
SDF-1: Stromal Cell Derived Facor 1
MIF: Macrophage Migration Inhibitory Facor
cAMP: Circular Adenosinemonophposphate
CXCR2: Chemokine Receptor 2
IP3: Inositol-1,4,5-trisphpsphat
JAK: Janus Kinase
STAT: Signal Transducer and Activator of Transcription
PI3K: Phosphoinositid-3-Kinase
ERK: Extracellular-signal Regulated Kinase
CTC: Circulating Tumor Cell
DCC: Disseminated Tumor Cell
RECIST: Response Evaluation Criteria In Solid Tumors
KPS: Karnofsky Perfomance Status
CNS: Central Nervous System
FCCS: Fluorescence Cross Correlation Spectroscopy
DAMP: Danger Associated Molecular Pattern
ACKR3: Atypical Chemokine Receptor Kinase 3
MRI: Molecular Resonance Imaging
GFAP: Glial Fibrillary Acidic Protein
WGA: Whole genome amplification
