# Supplementary material for: Isolated metastasis of an EGFR-L858R-mutated NSCLC of the meninges: the potential impact of CXCL12/CXCR4 axis in EGFRmut NSCLC in diagnosis, follow-up and treatment
Source: Oncotarget. 2018 Apr 10;9(27):18844–57. doi: 10.18632/oncotarget.24787 (PMC5922360; doi:10.18632/oncotarget.24787)
Supplement: Supplementary file 1 [file oncotarget-09-18844-s001.pdf]

Isolated metastasis of an EGFR-L858R-mutated NSCLC of the meninges: the potential impact of CXCL12/CXCR4 axis in $EGFR_{mut}$ NSCLC in diagnosis, follow-up and treatment

SUPPLEMENTARY MATERIALS

Liquid biopsy

Tumor cells were captured using anti-epithelial cell adhesion molecule (EpCAM)-antibody-bearing ferrofluid. The captured cells were also checked for cytokeratin positivity and absence of leukocyte common antigen CD45. The integrity of the nucleus was depicted with 40, 6-diamidino-2-phenylindole (DAPI) staining. Unlike for blood samples, the cerebrospinal fluid was run in the control mode of the instrument due to its transparent appearance. The captured cells were washed out from the CellSearch® cartridge with 1X PBS. The cells were then centrifuged for 5 min at 1,000 g and the pellet was re-suspended in 1X PBS.

The cell suspension was placed on a glass chamber for screening. Isolation of single cells was achieved manually using an inverted fluorescent microscope (Olympus; Hamburg; Germany) attached to a micromanipulator [1]. Single EpCAM-positive cells were picked using the glass capillary attached to the micromanipulator. Each isolated single cell, a cell pool and a picking control (negative control) were placed individually in Proteinase K containing buffer [2].

Whole genome amplification (WGA) was processed immediately after isolation of the cells using Ampli1TM WGA Kit (Silicon Biosystems, San Diego USA) according to the manufacturer's instructions. The whole genome was fragmented using MSE1 restriction enzyme followed by adaptor ligation and primary PCR amplification [3, 4].

The quality of the WGA products, Genome Integrity Index (GII), was tested using a 4-plex multiplex PCR [5]. For this purpose, 1 µl WGA template was used in 10 µl of mastermix containing 1× FastStart PCR Buffer (including 20 mM MgCl2), 200 nM dNTPs, 4 µg BSA, 0.4 µM of each primer (KRAS, D5S2117, TP 53 Exon 2/3 and CK19) and 0.5 U FastStart Taq Polymerase. PCR was started with at 95° C for 4 min, followed by 32 cycles of 95° C for 30 s, 58° C for 30 s and 72° C for 90 s, and a final elongation step of 7 min at 72° C. To determine the GII, PCR products were visualized using a 1.5 % agarose gel.

Sequencing of EGFR Exons 18, 19, 20 and 21 was accomplished on three DCCs with GII4. The PCR products were purified using Qiagen PCR Purification Kit (Qiagen GmbH, Hilden Germany) according to manufacturer's recommendations. The DNA concentration of the purified products were measured using NanoDrop ND-1000 instrument. A concentration of 5 ng/µl was adjusted for sequencing reaction which was outsourced from Eurofins Co (Munich, Germany).

Praparation of 68-Ga pentixafor

The eluate of the $^{68}Ge/^{68}Ga$ generator ($^{68}Ga^{3+}$ in 0.6 M HCl) was immobilized on a cation exchange cartridge, eluted with 5 M NaCl and added to a solution of 40 µg Pentixafor (CPCR4.2 trifluoroacetate, Scintomics GmbH, Germany), cyclic pentapetide ligand with high affinity for CXCR4, in HEPES-buffer. The labeling reaction took place at 125° C for 6 min. After cooling, the reaction mixture was transferred to the waste vial via a SepPak Light C18-Catridge (Waters, Germany). After rinsing with H2O to remove unreacted ^{68}Ga-Chloride the ^{68}Ga-labeled peptide was eluted with 2 ml of 50% ethanol solution, diluted with phosphate buffer solution and passed through a sterile filter (0.22 µm).

Prior use, the quality of ^{68}Ga-Pentixafor was assessed according to the standards described in the European Pharmacopeia for ^{68}Ga-Endotreotide (European Pharmacopeia, Monograph 01/2013:2482). Radiochemical purity was determined by gradient high performance liquid chromatography and a thin layer chromatography. Additionally, the product was tested for pH, radionuclide purity, sterility and endotoxins.

FDGPET/CT, 68-Ga-Pentixafor-PET/CT

FDG-PET/CT imaging was performed using a Biograph 16 PET/CT scanner (CTI-Siemens, Erlangen, Germany) consisting of a 16-slice multidetector CT (0.5 seconds per revolution) and a PET detector with an axial and transaxial field-of-view of 162 mm and 585 mm, respectively. After a fasting period of at least 4 hours, 3 MBq FDG per kilogram body weight were injected

intravenously. Blood glucose of diabetic patients was strictly controlled to be below 200 mg/dl. To increase renal tracer elimination an injection of 20 mg furosemide was given as well as oral hydration. To minimize muscular FDG uptake patients were advised to stay in a silent lying position. Warming blankets were used to avoid freezing of the patients and keep the tracer accumulation in brown fat to a minimum. Patients were instructed to void the bladder prior to scanning and to remove all metal parts. After a waiting period of 60 min post injection the PET/CT acquisition was performed in two parts with a supine position with the arms flanking the body for imaging the head and neck region as well as with an additional scan with elevated arms to acquire images of the trunc. Overlapping bed positions with 3 min PET acquisition time each were used. The same area was covered by a low-dose CT scan (tube current 50 mAs, tube voltage 120 kVp) without use of contrast agents.

PET images (slice thickness 5 mm) were corrected for random coincidences, decay, scatter and attenuation and reconstructed iteratively using the ordered subsets expectation maximization algorithm (OSEM) with 4 iterations and 8 subsets. PET images were scaled to allow standardized uptake value (SUV) measurements. With a region-of-interest technique the maximum SUV (SUVmax) was measured in the PET images by drawing an ROI around the most intense area of the tumor.

Ga-68-Pentixafor PET/CT was performed the same way with an activity of 1.5 MBq Ga-68-Pentixafor per kilogram body weight without fasting and without checking for blood glucose levels.

FCCS analysis

Membranes were prepared from a cell pellet corresponding to 2×10^{-7} cells by sonification in TBS/150 mM NaCl on ice. Cell debris was removed by low speed centrifugation and membranes harvested from the cleared lysate by subsequent centrifugation for 1 h at 21.000 g at 4° C. The receptors were solubilized by head over tail incubation in detergent mix of DDM (n-dodecyl β-D-maltoside)/CHAPS/CHS (cholesteryl hemisuccinate) at final concentrations of 0.25/0.5/0.1% (w/v), respectively. Unsolubilized membrane material was pelleted by centrifugation for 1 h at 100.000 g at 4° C. Solubilized GPCRs were directly used for FCCS binding assays.

FCCS measurements with samples at equilibrium (typically with a volume of 20 µl) were performed with a ConfoCor2 FCS unit connected to an Axiovert 100 M equipped with a C-Apochromat 40× water immersion lens, NA 1.2 (Carl Zeiss, Jena, Germany) whereas FCCS-data for binding kinetics were acquired on an Insight plate reader (Evotec Technologies, Hamburg, Germany) fitted with a U-Apo300 40× water immersion lens, NA 1.15 (Olympus, Germany). Data acquisition for samples in equilibrium typically took 20–60 s per sample. The kinetics was monitored by FCCS over the course of 20–60 min, during which the single measurements were taken for 5–20 s depending on the rate of the complex formation.

REFERENCES

1. Guzvic M, Braun B, Ganzer R, Burger M, Nerlich M, Winkler S, Werner-Klein M, Czyz ZT, Polzer B, Klein CA. Combined genome and transcriptome analysis of single disseminated cancer cells from bone marrow of prostate cancer patients reveals unexpected transcriptomes. Cancer Res. 2014; 74:7383–94. https://doi.org/10.1158/0008-5472.CAN-14-0934.
2. Imle A, Polzer B, Alexander S, Klein CA, Friedl P. Genomic instability of micronucleated cells revealed by single-cell comparative genomic hybridization. Cytometry A. 2009; 75:562–8. https://doi.org/10.1002/cyto.a.20733.
3. Klein CA, Schmidt-Kittler O, Schardt JA, Pantel K, Speicher MR, Riethmuller G. Comparative genomic hybridization, loss of heterozygosity, and DNA sequence analysis of single cells. Proc Natl Acad Sci U S A. 1999; 96:4494–9.
4. Klein CA, Seidl S, Petat-Dutter K, Offner S, Geigl JB, Schmidt-Kittler O, Wendler N, Passlick B, Huber RM, Schlimok G, Baeuerle PA, Riethmuller G. Combined transcriptome and genome analysis of single micrometastatic cells. Nat Biotechnol. 2002; 20:387–92. https://doi.org/10.1038/nbt0402-387.
5. Alberter B, Klein CA, Polzer B. Single-cell analysis of CTCs with diagnostic precision: opportunities and challenges for personalized medicine. Expert Rev Mol Diagn. 2016; 16:25–38. https://doi.org/10.1586/14737159.2016.1121099.

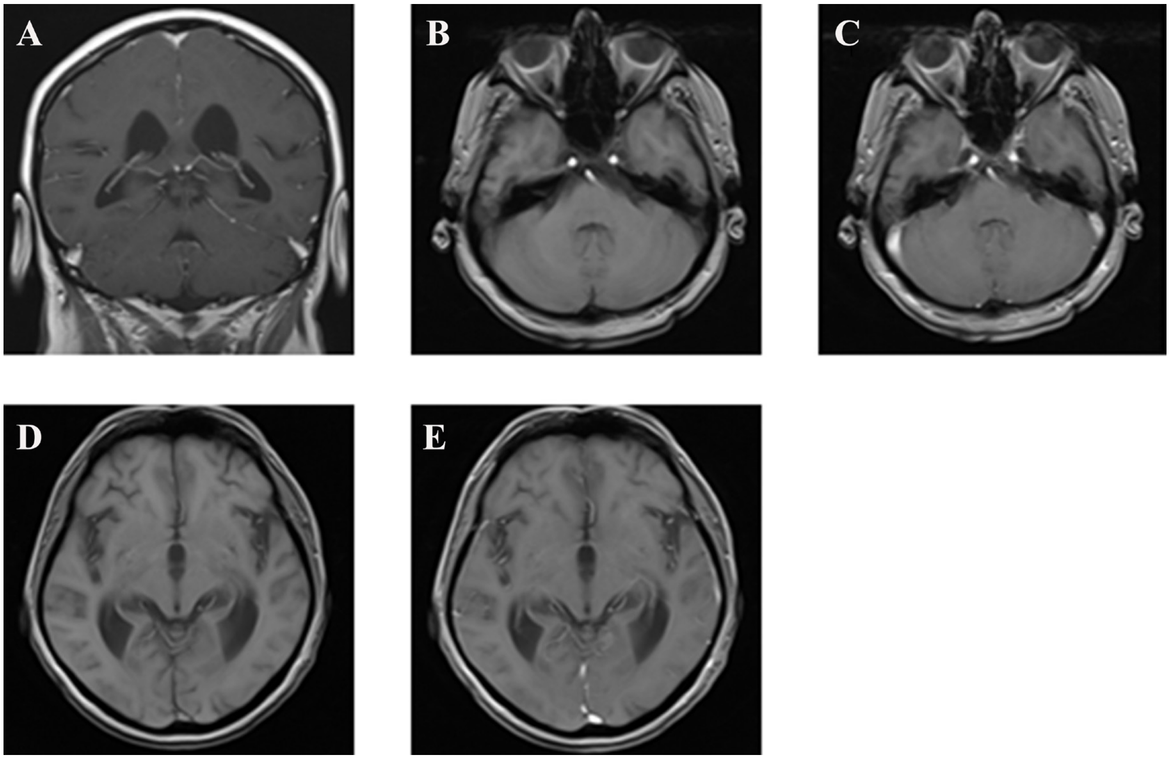

Supplementary Figure 1: Additional cranial MRI-imaging. (**A**) coronal T1-sequence after Gadovist showing no pathological meningeal enhancement; (**B**, **C**) axial T1-sequence non-contrast (B) and after Gadobutrol (C) showing no pathological meningeal enhancement (section below the representative section shown in Figure 1); (**D**, **E**) axial T1-sequence non-contrast (D) and after Gadobutrol (E) showing no pathological meningeal enhancement (section above the representative section shown in Figure 1).

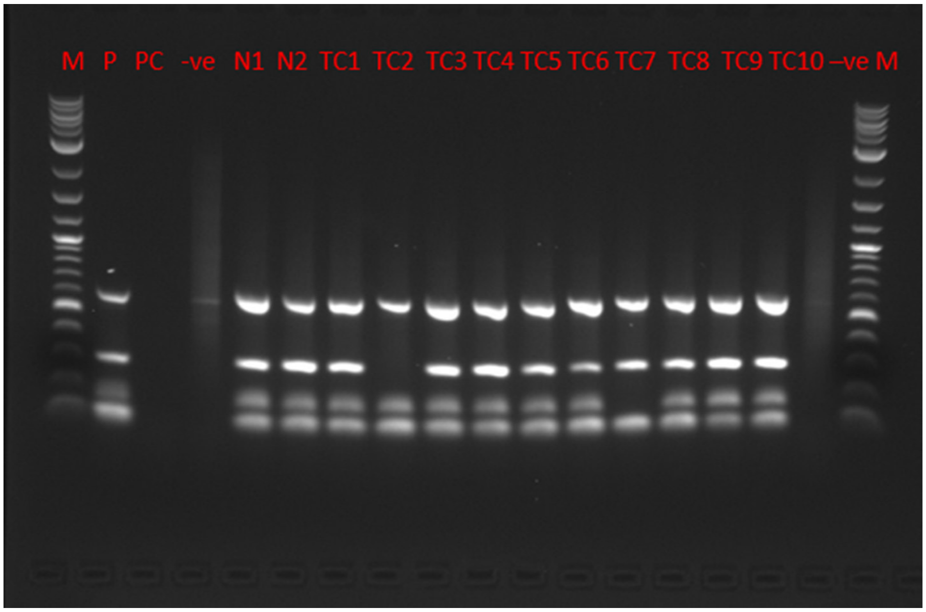

Supplementary Figure 2: Whole genome amplification: GII analysis of WGAs obtained from single DTCs through multiplex PCR. Except for cell number 2 and 7 all the isolated normal and tumor cells have GII4. Three random TCs with GII4 were selected for EGFR mutation analysis. M: 2-log DNA Ladder, P: positive control, PC: picking control, -ve: negative control N: normal cell, TC: tumor cell.
